# Effects of Tomato Root Exudates on *Meloidogyne incognita*

**DOI:** 10.1371/journal.pone.0154675

**Published:** 2016-04-29

**Authors:** Guodong Yang, Baoli Zhou, Xinyu Zhang, Zijun Zhang, Yuanyuan Wu, Yiming Zhang, Shuwen Lü, Qingdao Zou, Yuan Gao, Long Teng

**Affiliations:** 1 College of Horticulture, Shenyang Agricultural University, Shenyang, P. R. China; 2 Vegetable Research Institute, Liaoning Academy of Agricultural Sciences, Shenyang, P. R. China; James Hutton Institute, UNITED KINGDOM

## Abstract

Plant root exudates affect root-knot nematodes egg hatch. Chemicals in root exudates can attract nematodes to the roots or result in repellence, motility inhibition or even death. However, until recently little was known about the relationship between tomato root exudates chemicals and root-knot nematodes. In this study, root exudates were extracted from three tomato rootstocks with varying levels of nematode resistance: Baliya (highly resistant, HR), RS2 (moderately resistant, MR) and L-402 (highly susceptible, T). The effects of the root exudates on *Meloidogyne incognita* (*M*. *incognita*) egg hatch, survival and chemotaxis of second-stage juveniles (J2) were explored. The composition of the root exudates was analysed by gas chromatography/mass spectrometry (GC/MS) prior to and following *M*. *incognita* inoculation. Four compounds in root exudates were selected for further analysis and their allopathic effect on *M*. *incognita* were investigated. Root exudates from each tomato rootstocks (HR, MR and T strains) suppressed *M*. *incognita* egg hatch and increased J2 mortality, with the highest rate being observed in the exudates from the HR plants. Exudate from HR variety also repelled *M*. *incognita* J2 while that of the susceptible plant, T, was demonstrated to be attractive. The relative amount of esters and phenol compounds in root exudates from HR and MR tomato rootstocks increased notably after inoculation. Four compounds, 2,6-Di-tert-butyl-p-cresol, L-ascorbyl 2,6-dipalmitate, dibutyl phthalate and dimethyl phthalate increased significantly after inoculation. The egg hatch of *M*. *incognita* was suppressed by each of the compound. L-ascorbyl 2,6-dipalmitate showed the most notable effect in a concentration-dependent manner. All four compounds were associated with increased J2 mortality. The greatest effect was observed with dimethyl phthalate at 2 mmol·L^-1^. Dibutyl phthalate was the only compound observed to repel *M*. *incognita* J2 with no effect being detected in the other compounds. Each of the four compounds were correlated with a reduction in disease index in the susceptible cultivar, T, and tomato seedlings irrigated with L-ascorbyl 2,6-dipalmitate at 2 mmol·L^-1^ showed the best resistance to *M*. *incognita*. Taken together, this study provided a valuable contribution to understanding the underlying mechanism of nematode resistance in tomato cultivars.

## Introduction

The tomato is one of the most widely-cultivated crops in the world. In 2013, global yields reached 164 million tons (United Nations Food and Agriculture Organization (FAO) statistics; see URLs) and production value exceeded $55 billion [1]. Regular outbreaks of root-knot nematode disease caused by *M*. *incognita* have occurred in recent years, impacting considerably on both tomato crop yield and quality, and are an increasing problem in global tomato production [2–5].

In the 1940s, the nematode resistant gene *Mi* was identified and shown to be unique source of resistant in all tomato cultivars [6]. Shortly after, many tomato cultivars and rootstocks carrying the *Mi* gene emerged. This was regarded as the most safe and effective approach for the prevention of root-knot nematode disease [7–8]. Beyond the gene, *Mi*, the main target of researchers worldwide has become to understanding the allelopathic effect of root exudates on nematodes [9–14]. Root exudates can affect parasitic nematode egg hatch [15–19]. Infective nematode stages respond to plant signals originating from root exudates or sites of previous nematode penetration to find and recognize their hosts [20]. For example, carbon dioxide released from roots attracts *M*. *incognita* [21–22]. Tannic acids, flavonoids, glycoside, fatty acids and volatile organic molecules regulate attraction and repellence of second-stage juveniles (J2) for plant hosts [23–24]. Furthermore, plant-parasitic nematodes can be guided by terpenoids and volatile compounds induced by the actions of herbivores. For instance, *Tylenchulus semipenetrans* nematodes were found to be more attracted to *Citrus* spp. roots infested by weevil larvae than to intact plants [25]. However, until now little was known about the relationship between root exudate semiochemicals and the process by which root-knot nematodes find and recognize their hosts. In the 2011 International Symposium on Vegetable Grafting, one of the major themes was to reinforce research on soil-borne diseases including blight, root-knot nematode and root rot, and to enhance rootstock resistance [26]. Early research assessed the resistance of twelve tomato rootstocks to *M*. *incognita* [27]. Thus, there is a clearly recognized need for systematic research on the relationship of tomato rootstock and resistance to root-knot nematode.

In the current study, root exudates from three rootstocks including Baliya (highly resistant, HR), RS2 (moderately resistant, MR) and L-402 (highly susceptible, T) were investigated. The effects of the root exudates on egg hatch of *M*. *incognita*, survival and chemotaxis of the second-stage juvenile (J2) were assessed. Constituent change in exudates pre- and post-inoculation was evaluated to identify any potential compositions specifically related to nematode resistance. Finally, in vitro and field tests were performed to explore the allelopathic effects of root exudates on *M*. *incognita*. The results of the study add to a growing body of important literature illustrating the effect of root exudates on *M*. *incognita*.

## Materials and Methods

### Tomato materials

Three tomato rootstocks previously assessed for nematode resistance [19] were selected. Baliya (highly resistant, HR) seeds were supplied by Japan TAKII co., LTD while RS2 (moderately resistant, MR) and L-402 (highly susceptible, T) seeds were procured from China Liaoning Horticultural Seedlings Co., LTD. The tomato rootstocks were sown in May 2014 and at 2-leaf stage were transplanted in plastic pots (18 cm × 18 cm) filled with a steam-sterilized sand and top soil mix (ratio 1:2). 60 seedlings were maintained for each variety in a greenhouse. 20 seedlings of each variety were used for the bioassay of nematode egg hatch, survival and chemotaxis of J2. Root exudates (see below for details) obtained from the root systems at four weeks of growth (bud stage). The remaining plants were subdivided into two equal groups with 20 seedlings from each variety in each. The first of these were inoculated with J2 at the 4-leaf stage of growth and the second were used as a control and not inoculated at all. At 40 days post-inoculation, root exudates obtained from the root systems of both the treatment and control groups seedlings were used for GC-MS analysis (described below).

### Meloidogyne incognita

*M*. *incognita* specimens were donated by Institute of Plant Protection, Shenyang Agricultural University. They were maintained as a greenhouse stock culture on tomato cv. L-402. Infected plants were uprooted, and the entire root system was dipped in water and washed gently to remove adhering soil. Egg masses of *M*. *incognita* were collected using forceps, disinfected with a 0.4% solution of NaClO for three minutes then rinsed three times with sterilized water. Egg masses were placed in an autoclaved tissue grinder and gently crushed, then pipetted onto an autoclaved 25 μm aperture sieve and rinsed with sterilized water. A subset of the collected eggs (see below for full details) was used for the egg hatch bioassay whilst the remainder was incubated at 25°C until hatched in order to obtain J2. The fresh nematode inoculum (2,000 J2·ml^-1^) was used for in vitro and pot experiments.

### Collection of root exudates and extract preparation

Following the method used by Wang et al. (2005) [28], six tomato seedlings of each variety were carefully removed from the block and washed gently. They were then transferred to a 500ml Erlenmeyer flask with 500mL of sterilized distilled water, shaded, aerated and maintained within the temperature range 23–25°C. After 10 hours, the distilled water was collected and filtered. This was repeated five times, after which 20ml of the filtrate was used for the bioassay, and the remaining amount was concentrated to 20 ml with a vacuum rotary evaporator before being transferred to a separating funnel with an equivalent volume of absolute ether. After the ether fraction was collected, the previous step was repeated three times. Finally, the ether fractions obtained were combined, concentrated to a volume of 2 ml with a vacuum rotary evaporator, and then dried. A small quantity of absolute Na_2_SO_4_ was added and the solutions were allowed to evaporate naturally to 1.0ml in a drying cabinet at 25°C.

### GC-MS analysis

The chemical constituents of root exudates were detected by 6890-5973N gas chromatograph /mass spectrometer (Agilent). Samples were introduced via a TG-5MS capillary column of 30m × 0.25μm ×250μm. The column temperature was initially held at 50°C for 4 min, then programmed to 250°C at a rate of 6°C ·min^-1^, with a final hold time of 15 min. Helium was used as the carrier gas with a linear velocity of 1ml·min^-1^; the injection volume was 1μL. The ionization voltage and temperature in the electron impact (El) mode were 70 eV and 230°C respectively. Quadrupole temperature was 150°C, and the scanned range was 35–520 m·Z^-1^. The compounds were identified by comparison of retention times and mass spectral data. Relative contents of constituents were calculated by the resultant method, and their name determined according to the standard map (spectrogram data-base NIST98).

### Relative suppression rate of *M*. *incognita* egg hatch

A 24-well tissue culture plate was used for the *M*. *incognita* egg hatch assay. An autoclaved 25 μm aperture sieve (1 cm diam., 1 cm high) was placed in each well. Approximately 200 eggs were transferred onto the sieve and then 1.0 ml of root exudate was added in each well. The culture plates were transferred to an incubator set at 25°C. As a control, 1.0 ml of sterilized water was added in each well. The experiment was repeated three times. On Day 3 the number of J2 juveniles in each well was recorded after the sieves with eggs were transferred to the new culture plates filled with the corresponding test solutions, then recorded and refreshed root exudates once a day. After five incubation periods, the total number of J2 was calculated.

The relative suppression rate was calculated as follows:

Relative suppression rate (%) = (number of J2 in sterilized water—number of J2 in root exudate) / number of J2 in sterilized water × 100

### Corrected mortality rate of *M*. *incognita* J2

Aliquots (50 μl) of J2 suspension liquid (about 100 J2) were placed in a well with 1.0ml of root exudate (sterilized water as control), cultivated in a constant temperature incubator at 25°C. This was repeated three times. The motility of J2 was monitored by viewing with a dissecting microscope once every 6 hours. At last, the mortality rate of J2 was assessed by the eyelash needle stimulus method at 24 h and 48 h [29].

Corrected mortality rate (%) = (mortality rate of J2 in root exudate—mortality rate of J2 in sterilized water) / (1- mortality rate of J2 in sterilized water) ×100

### Chemotaxis of *M*. *incognita* J2

**Fig 1:**
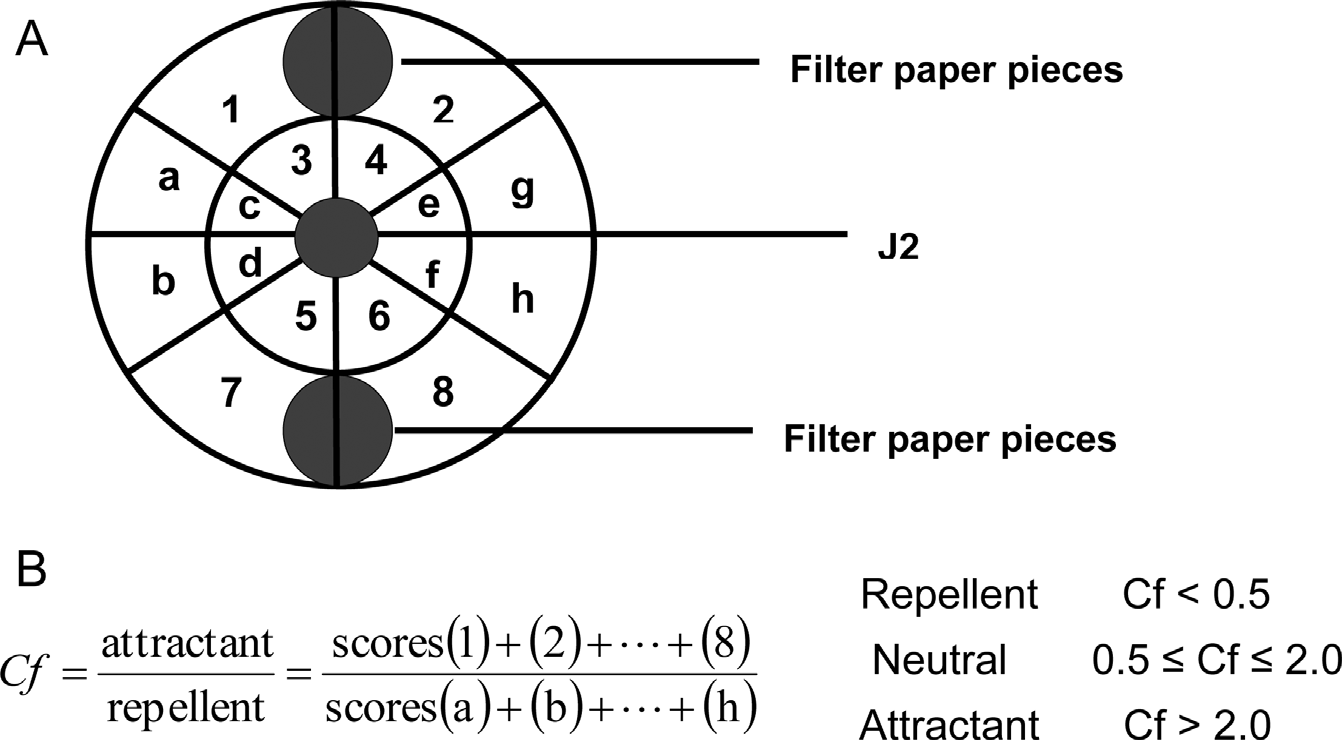
Diagram of *in vitro* chemotaxis assay. (A) Grid for the analysis of nematode preferential orientation on the medium; each segment of the grid was given a score for the presence (1) or absence of nematode tracks (0); (B) Calculation of the chemotaxis factor (Cf).

Using a modified method of Wuyts et al. [30], 5 cm petri dishes divided into 16 segments were filled with 5 ml water agar (0.4%). After solidification of the culture medium, filter paper pieces (1 cm diam.) dipped in different root exudates were placed on the opposite sides of the dish (Fig 1). After 1 hour, twenty *M*. *incognita* J2 were pipetted onto the medium in the middle of the plate in a minimal volume of water. J2 were allowed to move over the medium for 3 hours at 25°C constant temperature in the dark. Afterwards, J2 movement was stopped by spraying with ethanol (70%), and the locations of J2 were recorded by stereomicroscope. Control treatments used sterilized water instead of root exudates and four replicates.

### Allelopathic effect of simulated components on *M*. *incognita* in vitro

On the basis of component analysis of root exudates from the highly, moderately resistant and highly susceptible tomato plants, four components including of 2,6-di-tert-butyl-p-cresol (A), L-ascorbyl 2,6-dipalmitate (B), dibutyl phthalate (C)and dimethyl phthalate (D) were selected to be tested at four concentrations (0.0, 0.5, 1.0 and 2.0 mmol·L^-1^). These compounds were first dissolved in absolute ethyl alcohol, then distilled water was added slowly to achieve the required concentration; the final concentration of ethanol was 1.0%, and controls (0.0 mmol·L^-1^) were 1.0% ethanol. Effect of simulated components on egg hatch, survival and chemotaxis of J2 were determined using the methods described above.

### Allelopathic effect of simulated components on *M*. *incognita* in field

Tomato cv.L-402 (T) was sown in September 2014 and when at the 2-leaf stage, as described above, was transplanted to plastic pots (18 cm × 18 cm) filled with steam sterilized soil. After five days, 20 seedlings (one treatment) were irrigated respectively with 50 ml each of the components described above at four concentrations (0.0, 0.5, 1.0 and 2.0 mmol·L^-1^) once every 3-days. Each treatment was replicated three times. Three days after the third irrigation, each seedling was inoculated with 3,000 J2. Forty days after inoculation, seedlings were removed from the pots and root knot rates and disease index of roots were investigated according to previously described evaluation and classification standards [31]. Briefly, 0 grade, root without root knot; 1 grade, root with 1%–20% root knots; 2 grade, root with 21%–40% root knots; 3 grade, root with 41%–60% root knots; 4 grade, root with 61%– 80% root knots; 5 grade, root with 81%–100% root knots.

Diseaseindex(DI)=∑(numberofseedlings×correspondinggrade)/(totalnumberofseedlings×topgrade)×100

### Statistical analysis

Bioassay data were analyzed using the DPS software (Refine Information Tech. Co., Ltd, China), the significant differences among treatments were determined by Duncan's new multiple range method at P < 0.05 and P < 0.01.

## Results

### Effects of root exudates on *M*. *incognita*

#### Root exudates effect on *M*. *incognita* egg hatch

**Fig 2:**
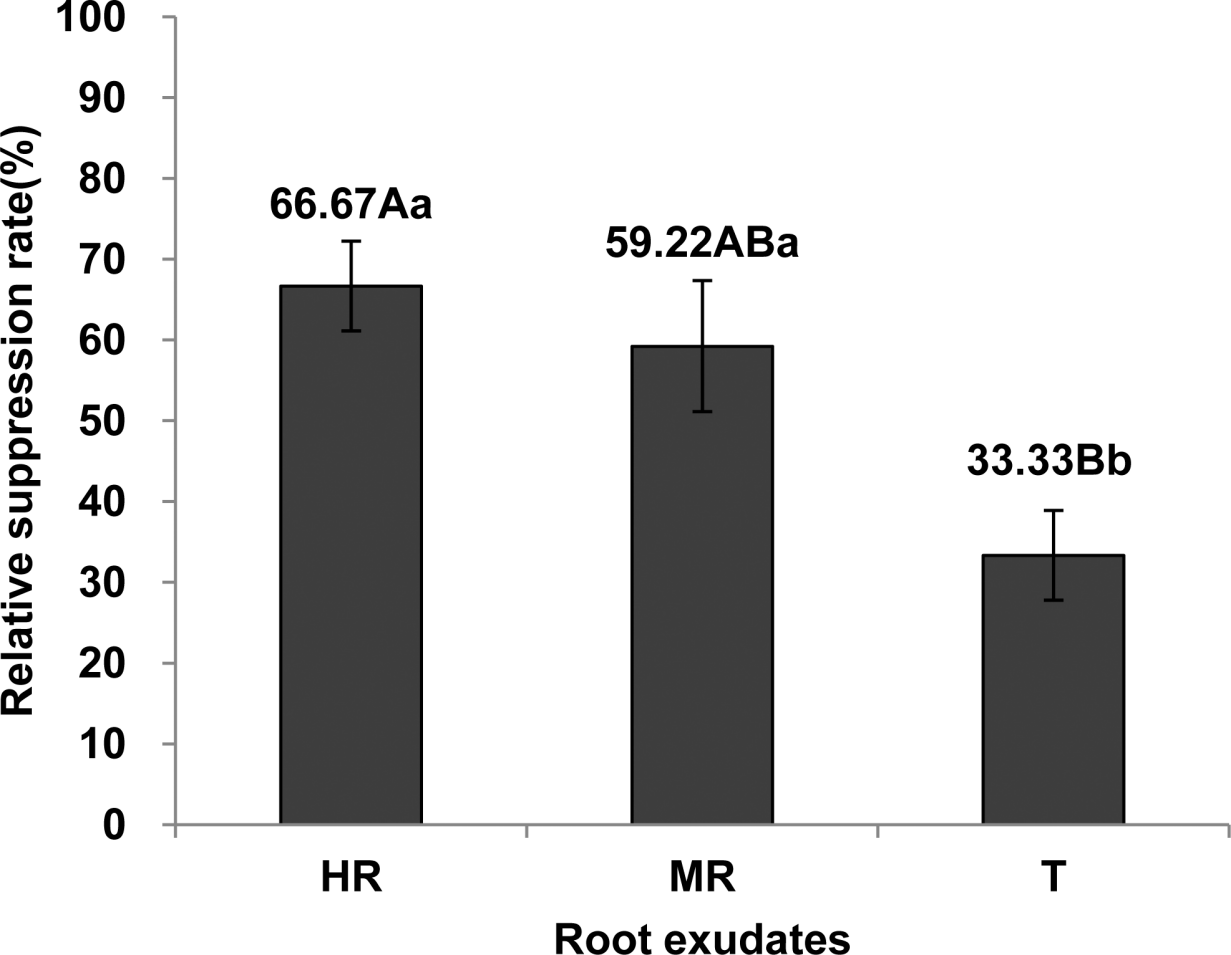
Effects of root exudates on *M*. *incognita* egg hatch. HR, MR and T represent the root exudates from three tomato strains: Baliya (highly resistant), RS2 (moderately resistant) and L-402 (highly susceptible), respectively. Total number of J2 in each root exudate (sterilized water as control) was calculated at 8d after starting the assay. Relative suppression rate (%) = (number of J2 in sterilized water—number of J2 in root exudate) / number of J2 in sterilized water × 100. Each bar represents the mean, and error bars indicate standard error of the mean from three replicates. Capital and lower case letters indicate significant group differences at the levels of 0.01 and 0.05, respectively.

The root exudates from the three tomato rootstocks (HR, R and T strain) were found to suppress egg hatch of *M*. *incognita* (Fig 2). The suppression rate of the exudates from high resistance (HR) strain, Baliya (66.67%) was similar to that of the moderate resistance (MR) strain, RS2 (59.22%). Both were significantly higher than from the susceptible (T) strain, L-402 (33.33%) (P < 0.05). At the end of hatch assay, root exudates were diluted 2 times in distilled water, and unhatched eggs were incubated again for 7 days to check the reversibility of hatch inhibition. The result also proved that inhibiting effect hadn’t been weakened obviously by the dilution of root exudates (S1 Fig).

#### Root exudates effect on *M*. *incognita* J2 survival

**Fig 3:**
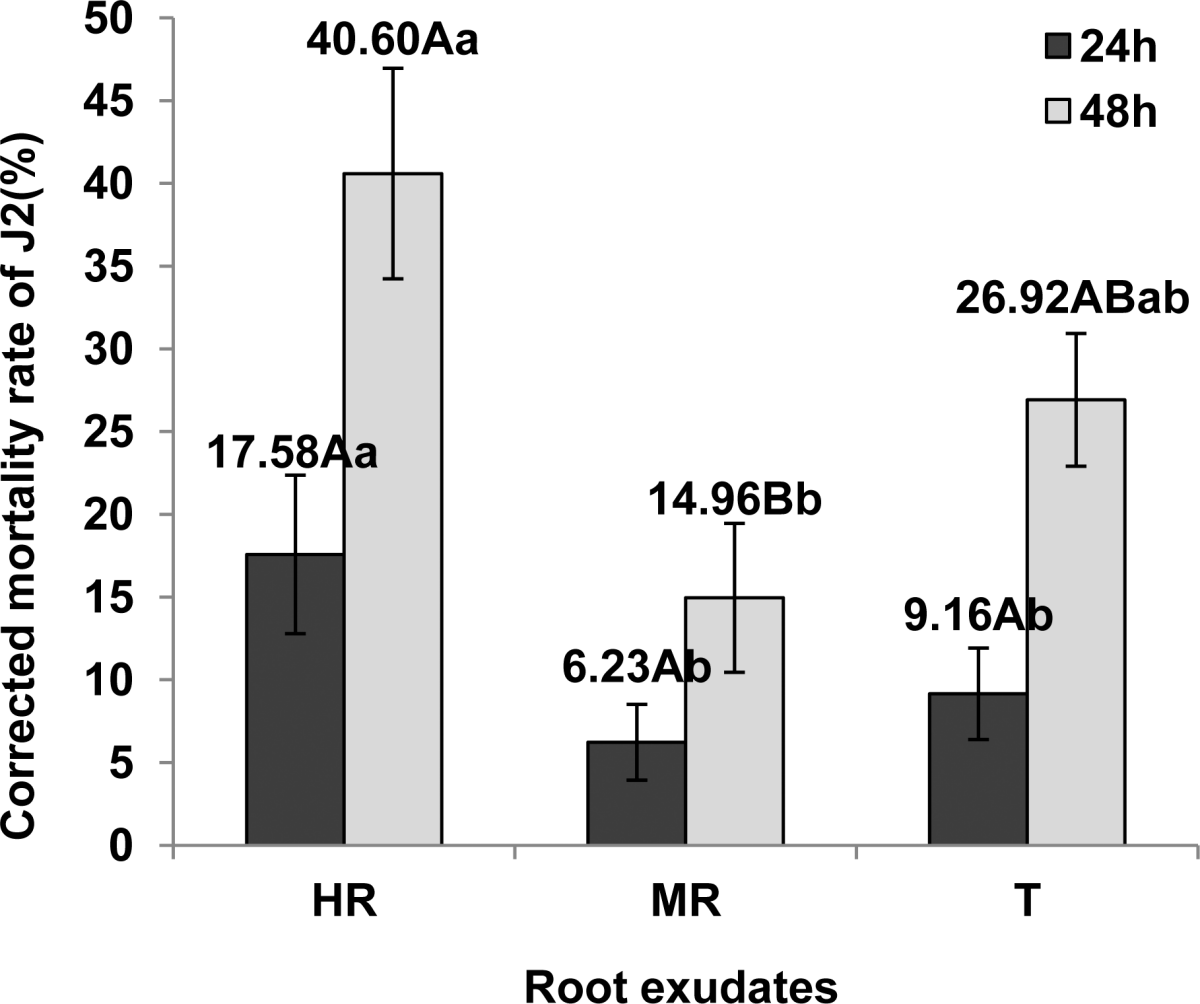
Effects of root exudates on corrected mortality of *M*. *incognita* J2. HR, MR and T represent the root exudates from three tomato strains: Baliya (highly resistant), RS2 (moderately resistant) and L-402 (highly susceptible), respectively. The mortality of J2 in each root exudate (sterilized water as control) was assessed by the eyelash needle stimulus method at 24 h and 48 h after starting the assay. Corrected mortality rate (%) = (mortality rate of J2 in root exudate—mortality rate of J2 in sterilized water) / (1—mortality rate of J2 in sterilized water) ×100. Each bar represents the mean, and error bars indicate standard error of the mean from three replicates. Capital and lower case letters indicate significant group differences at the levels of 0.01 and 0.05, respectively.

The corrected mortality rate of *M*. *incognita* J2 exposed to root exudate from Baliya (HR) after 24 hous was 17.58%. *M*. *incognita* J2 exposed to root exudates from RS2 (MR) and L-402 (T) showed lower mortality rate than in the HR treatment (6.23% and 9.16%, respectively) (P < 0.05). As shown in Fig 3, the corrected mortality rate of J2 exposed to root exudate from Baliya (HR) at 48 hours was 40.60%, still significantly higher than RS2 (MR) (14.96%) and L-402 (T) (26.92%) (P < 0.05). Furthermore, the reversible nematostatic effect was found after 6 hours. The quiescence of J2 was observed obviously with treatment of root exudates from Baliya (HR) and L-402 (T) and recovered before 18 hours (S2 Fig). Therefore, we confirmed the J2 death by the eyelash needle stimulus method at 24 hours and 48 hours.

#### Root exudates effect on chemotaxis of *M*. *incognita* J2

As shown in Table 1, the root exudate from HR strain was repellent to J2 while that from the MR strain was neutral. The root exudates from T strain and sterilized water (control) attracted *M*. *incognita* J2.

**Table 1:** Effects of root exudates on chemotaxis of *M*. *incognita* J2 at WA plate.

Root exudates^a^	Cf value^b^		Chemotaxis^c^
**HR**	0.45± 0.17	Ab	repellent
**MR**	1.56±0.97	Aab	neutral
**T**	2.96±1.25	Aa	attractant
**CK**	2.21±1.31	Aab	attractant

^a^ HR, MR and T represent the root exudates from three tomato strains: Baliya (highly resistant), RS2 (moderately resistant) and L-402 (highly susceptible), respectively; CK, sterilized water.

^b^ Cf, chemotaxis factor; Values represent the means ± standard deviation (SD) (n = 4); Capital and lower case letters indicate significant group differences at the levels of 0.01 and 0.05, respectively.

^c^ Attractant Cf >2.0, repellent Cf < 0.5, neutral 0.5 ≤ Cf ≤ 2.0.

### Allelopathic effects of simulated components on *M*. *incognita*

#### Component analysis of root exudates

The main chemical components of the root exudates were hydrocarbons compounds, comprising more than 59% of the total, with no difference detected pre- and post-inoculation. Esters were the second most abundant set of compounds, ranging between 6.96% and 16.91%. Phenol compounds accounted for less than 5% of the total. The relative contribution of both esters and phenols (Table 2) increased post-inoculation (P < 0.05). In ester and phenol compounds, the relative amount of, L-ascorbyl 2,6-dipalmitate, dibutyl phthalate, dimethyl phthalate and 2,6-Di-tert-butyl -p-cresol changed post-inoculation. Dibutyl phthalate, dimethyl phthalate and 2,6-Di-tert-butyl-p-cresol increased significantly (P < 0.01), with the greatest increase being observed in root exudates from Baliya (HR). In the HR and MR strains, the amount of L-ascorbyl 2,6-dipalmitate increased significantly (P < 0.01), but in the T strain, it declined sharply (p < 0.01) after inoculation (Table 2).

**Table 2:** Component identified from tomato plant root exudates.

Name of the components	Relative content %					
	Inoculation			Without inoculation		
	HR	MR	T	HR	MR	T
**Hydrocarbons**	**67.45**	**59**	**65.58**	**61.02**	**64.47**	**71.66**
Dodecane	0.97	0.56	0.89	0.65	0.39	0.47
Heptadecane	2.45	2.87	1.86	2.41	2.87	6.52
Tridecane	0.48	0.42	0.36	0.35	0.31	0.46
Dodecamethylcyclohexasiloxane	-	-	-	-	-	0.29
2-Methyltridecane	0.22	0.23	0.2	0.16	0.18	-
2,6,10,14-Tetramethyl hexadecane	0.33	0.33	0.28	0	0	0.31
Tetradecane	1.75	1.67	1.57	1.32	1.24	1.38
4-cyclohexyl dodecane	0.27	0.24	0.26	0.22	0.21	0.29
Hexadecane	0.7	0.71	0.63	0.58	0.57	0.78
Docosane	0.38	0.38	0.39	0.31	0.29	0.49
Pentadecane	2.05	2.06	1.99	1.69	1.7	2.57
Heptacosane	0.28	0.96	0.77	1.12	2.03	0.44
1-Hexyl-3-methylcyclopentane	0.51	0.55	0.44	-	-	0.66
2-Methyl pentadecane	0.5	0.48	0.43	0.46	0.42	0.74
3-Methyl pentadecane	0.32	0.33	0.28	0.25	0.21	0.39
Hexatriacontane	0.19	0.25	-	-	0.19	0.41
2,6,10- Tetramethyl pentadecane	1.11	1.46	0.9	1.23	1.4	3.27
2-Tetramethyl hexadecane	0.46	0.68	0.33	0.39	0.42	0.95
2,6,10,14-Tetramethyl pentadecane	1.48	2.17	1.17	1.86	2.4	5.3
Octadecane, 2-methyl-	0.3	0.49	0.28	0.33	0.28	1.22
Octadecane	0.97	1.66	0.86	1.01	1.4	3.5
2,6,10,14-Tetramethyl hexadecane	0.92	1.6	0.87	1.14	1.68	3.83
Nonadecane	0.42	1.12	0.48	0.95	0.88	1.92
Eicosane	0.22	0.71	0.28	0.88	0.96	0.64
Heneicosane	0.28	0.56	0.25	0.69	1.15	0.79
1,54-dibromo-Tetrapentacontane	-	0.31	0.19	-	-	-
Tetracosane	0.43	1.92	0.81	0.98	2.48	0.63
Pentacosane	0.67	2.81	0.97	1.21	3.31	1.32
1-Octadecene	0.26	1	0.42	0.31	0.27	0.26
**Ketones**	**0.61**	**1.23**	**0.89**	**0.59**	**0.67**	**1.08**
7,9-Di-tert-butyl-1-oxaspiro (4,5)deca-6,9-diene-2,8-dione	**0.61**	**1.23**	**0.89**	**0.59**	**0.67**	**1.08**
**Esters**	**16.91**	**14.44**	**12.53**	**6.96**	**6.84**	**10.42**
Dimethyl phthalate	2.08	1.51	1.45	0.95	0.85	0.93
Diisobutyl phthalate	0.44	0.77	0.54	0.42	0.38	0.55
L-ascorbyl 2,6-dipalmitate	6.59	1.46	0.63	1.37	0.88	3.12
Methyl hexadecanoate	0.35	1.07	3.47	-	1.2	3.36
Dibutyl phthalate	5.8	3.41	2.26	1.91	1.38	1.22
Diisooctyl phthalate	1.65	6.22	4.18	2.31	2.15	1.24
**Phenols**	**3.25**	**2.86**	**3.64**	**1.37**	**1.86**	**2.75**
Guaiacol	0.27	0.35	0.19	0.19	0.12	-
Methyl eugenol	0.25	0.28	0.26	0.23	0. 24	0.26
2,4-Di-tert-butylphenol	0.38	0.66	0.6	-	0.37	0.46
2,6-Di-tert-butyl -p-cresol	1.25	1.11	0.68	0.64	0.41	0.73
2,2'-Methylenebis(6-tert-butyl-4-methylphenol)	1.1	0.77	1.91	0.31	0.96	1.3
**Phenolic acids**	**0.19**	**2.87**	**0.77**	**0.22**	**0.79**	**0.84**
Phenol	0.19	-	0.19	-	-	-
3,5-Di-tert-butylbenzoic acid	-	0.39	-	-	0.17	-
MYRISTIC-1-13C ACID	-	0.23	-	-	-	0.56
Palmitic acid	-	2.25	0.58	0.22	0.62	0.28
** Amines**	**0.17**	**1.13**	**0.83**	**-**	**-**	**0.29**
N,N'-Diisopropylbenzene-1,4-di​amine	0.17	-	0.2	-	-	-
p-Toluenesulfonamide	-	0.83	0.46	-	-	0.29
o-Toluenesulfonamide	-	0.3	0.17	-	-	-
**Aromatic hydrocarbons**	**0.59**	**-**	**0.17**	**0.19**	**-**	**-**
2,7-Dimethylnaphthalene	0.25	-	0.17	0.19	-	-
Benzocycloheptatriene	0.17	-	-	-	-	-
**Alcohols**	-	1.27	1.9	1.01	0.88	2.22
2-Butanol	-	-	0.26	-	-	0.48
2,3-Butanediol	-	1.27	1.64	1.01	0.88	1.74
**Others**	10.83	17.2	13.69	28.64	24.49	10.74
1-(4-Methoxymethyl-2,6-dimethylphenyl)ethanone	0.22	-	0.23	-	-	-
9-Acetylanthracene	0.17	-	0.18	0	-	-
2,4-Bis[(trimethylsilyl)oxy]benzoic acid trimethylsilyl ester	-	-	-	-	-	0.77

HR, MR and T represent the root exudates from three tomato strains: Baliya (highly resistant), RS2 (moderately resistant) and L-402 (highly susceptible), respectively.

#### Effects of simulated components on *M*. *incognita* egg hatch

**Fig 4:**
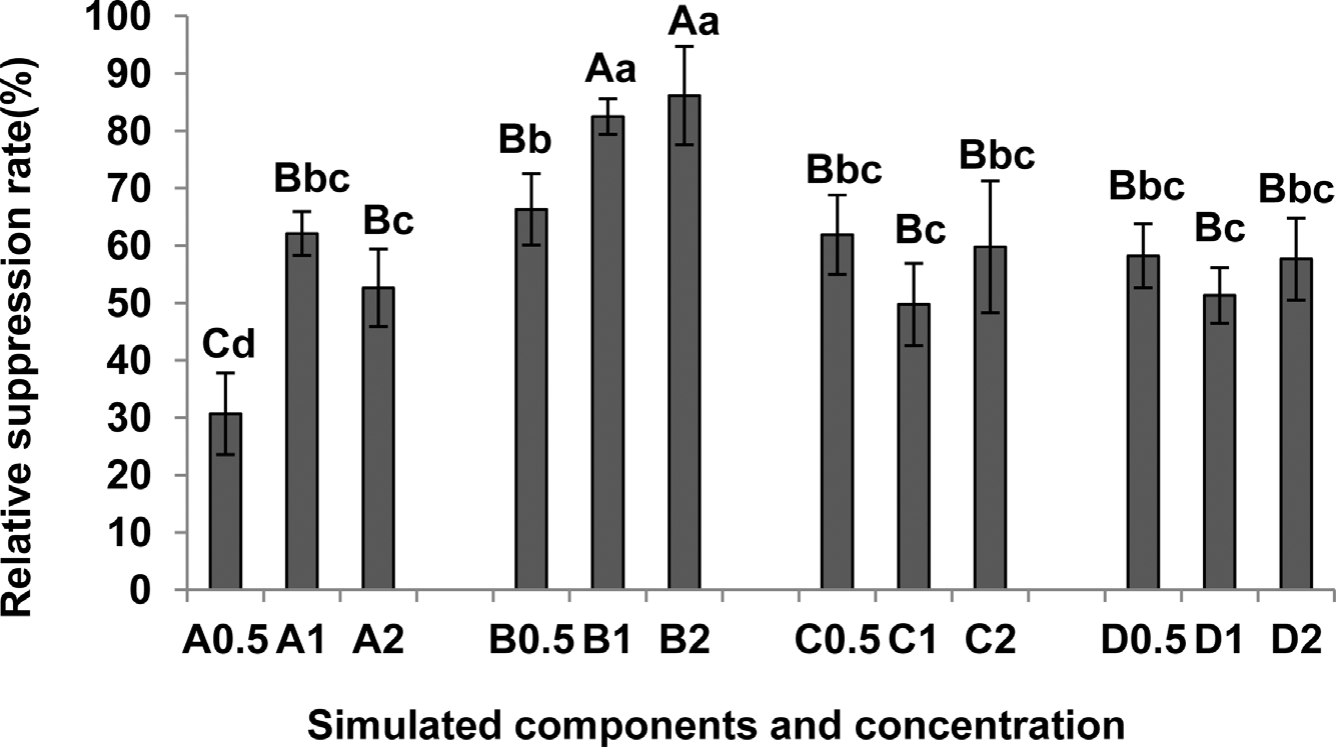
Effects of simulated components on relative suppression of *M*. *incognita* egg hatch. A, 2,6-Di-tert-butyl-p-cresol; B, L-ascorbyl-2,6-dipalmitate; C, dibutyl phthalate; D, dimethyl phthalate; 0.5,0.5 mmol·L^-1^; 1,1 mmol·L^-1^; 2,2 mmol·L^-1^. Total number of J2 in each treatment (1.0% ethanol as control) was calculated at 8d after starting the assay. Relative suppression rate (%) = (number of J2 in control—number of J2 in treatment) / number of J2 in control × 100. Each bar represents the mean, and error bars indicate standard error of the mean from three replicates. Capital and lower case letters indicate significant group differences at the levels of 0.01 and 0.05, respectively.

Four compounds all suppressed egg hatch of *M*. *incognita* (Fig 4; S1 Table) and the effect observed in 2,6-di-tert-butyl-p-cresol and L-ascorbyl 2,6-dipalmitate increased with concentration. L-ascorbyl 2,6-dipalmitate (B) suppressed the most, with the relative suppression rate reaching 86.15% at 2 mmol·L^-1^. It was significantly higher than the efficiency of the other compounds at all concentrations (P < 0.01).

#### Effects of simulated components on *M*. *incognita* J2 survival

**Fig 5:**
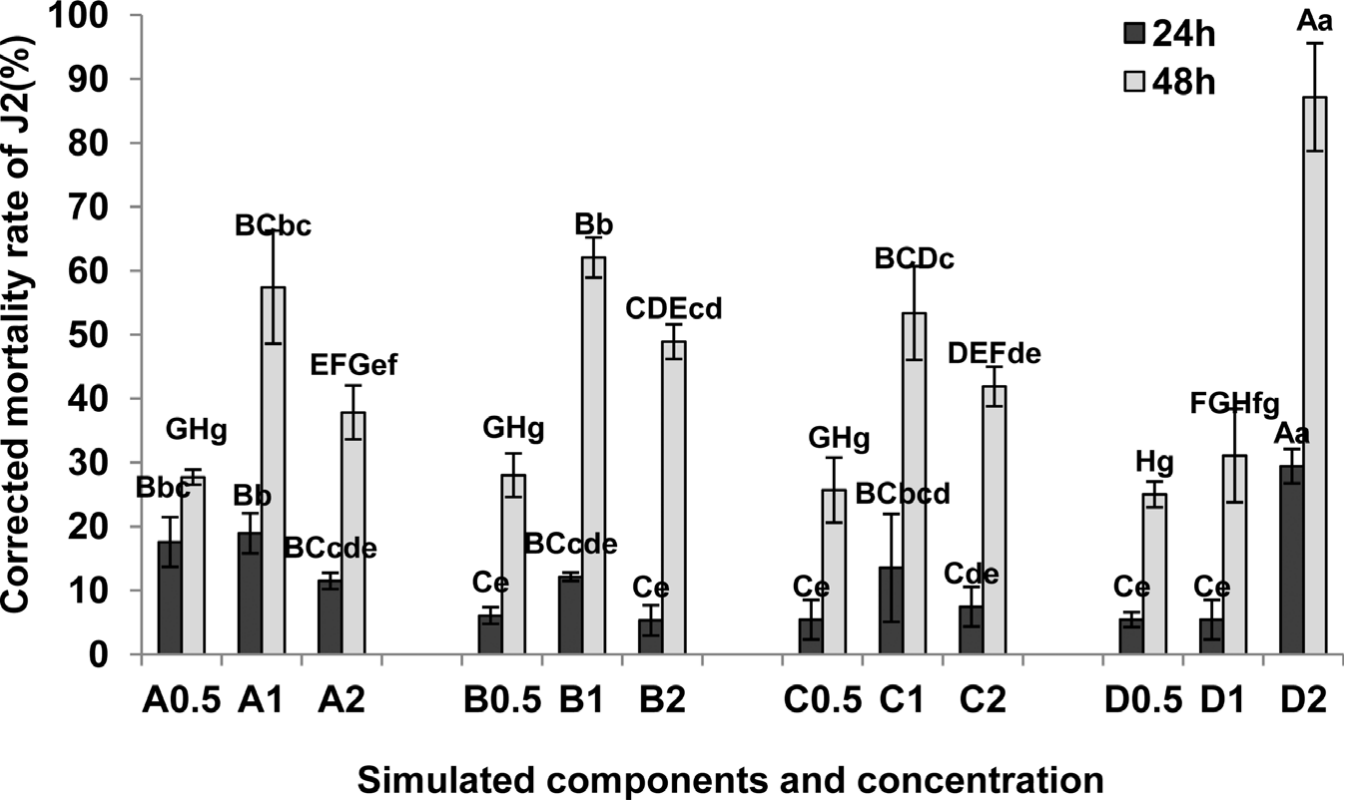
Effects of simulated components on corrected mortality of *M*.*incognita* J2. A, 2,6-Di-tert-butyl-p-cresol; B, L-ascorbyl-2,6-dipalmitate; C, dibutyl phthalate; D, dimethyl phthalate; 0.5,0.5 mmol·L^-1^; 1,1 mmol·L^-1^; 2,2 mmol·L^-1^. The mortality of J2 in each treatment (1.0% ethanol as control) was assessed by the eyelash needle stimulus method at 24 h and 48 h after starting the assay. Corrected mortality rate (%) = (mortality rate of J2 in treatment—mortality rate of J2 in control) / (1—mortality rate of J2 in control) ×100. Each bar represents the mean, and error bars indicate standard error of the mean from three replicates. Capital and lower case letters indicate significant group differences at the levels of 0.01 and 0.05, respectively.

The four exudate compounds all increased the mortality rate of *M*. *incognita* J2, but large differences were observed between the different concentrations (Fig 5; S2 Table). At 24 hours, the corrected mortality rates of J2 were highest with the 1mmol·L^-1^ of 2,6-Di-tert-butyl -p-cresol (A), L-ascorbyl 2,6-dipalmitate (B) and dibutyl phthalate (C) respectively. However, the corrected mortality rate at 2 mmol·L^-1^ of dimethyl phthalate (D) was significantly higher than the other compounds (P < 0.01). The corrected mortality rates of J2 followed a similar trend at 48 hours. The corrected mortality rate at 2 mmol·L^-1^ of dimethyl phthalate (D) increased from 29.42% up to 87.16%.

#### Effects of simulated components on chemotaxis of *M*. *incognita* J2

As shown in Table 3, only dibutyl phthalate (C) repelled *M*. *incognita* J2. All the other compounds were associated with a neutral response. The chemotaxis factor (Cf) value of 2,6-Di-tert-butyl-p-cresol (A) and CK were above 1.5 and the Cf values of the other compounds were below 1 (except for L-ascorbyl 2,6-dipalmitate at the 2 mmol·L^-1^). The ascending order according to the Cf value was C < B < D < CK < A.

**Table 3:** Effects of four simulated components on chemotaxis of *M*. *incognita* J2 at WA plate.

Treatment^a^	Cf value^b^		Chemotaxis^c^
CK	1.58±0.55	Aa	neutral
A0.5	1.79±0.66	Aa	neutral
A1	1.79±0.66	Aa	neutral
A2	1.67±0.77	Aa	neutral
B0.5	1.06±0.90	ABb	neutral
B1	0.53±0.33	Bbc	neutral
B2	0.69±0.23	Bbc	neutral
C0.5	0.49±0.12	Bbc	repellant
C1	0.40±0.20	Bc	repellant
C2	0.30±0.09	Bc	repellant
D0.5	0.84±0.19	Bbc	neutral
D1	0.82±0.47	Bbc	neutral
D2	0.69±0.24	Bbc	neutral

^a^ A, 2,6-Di-tert-butyl -p-cresol; B, L-ascorbyl 2,6-dipalmitate; C, Dibutyl phthalate; D, Dimethyl phthalate; 0.5,0.5 mmol·L^-1^; 1,1 mmol·L^-1^; 2,2 mmol·L^-1^;CK, 1.0% ethanol.

^b^ Cf, chemotaxis factor; Values represent the means ± standard deviation (SD) (n = 4); Capital and lower case letters indicate significant group differences at the levels of 0.01 and 0.05, respectively.

^c^ Attractant Cf >2.0, repellent Cf < 0.5, neutral 0.5 ≤ Cf ≤ 2.0.

#### Effects of simulated components on the disease index of tomato cv. L-402

**Fig 6:**
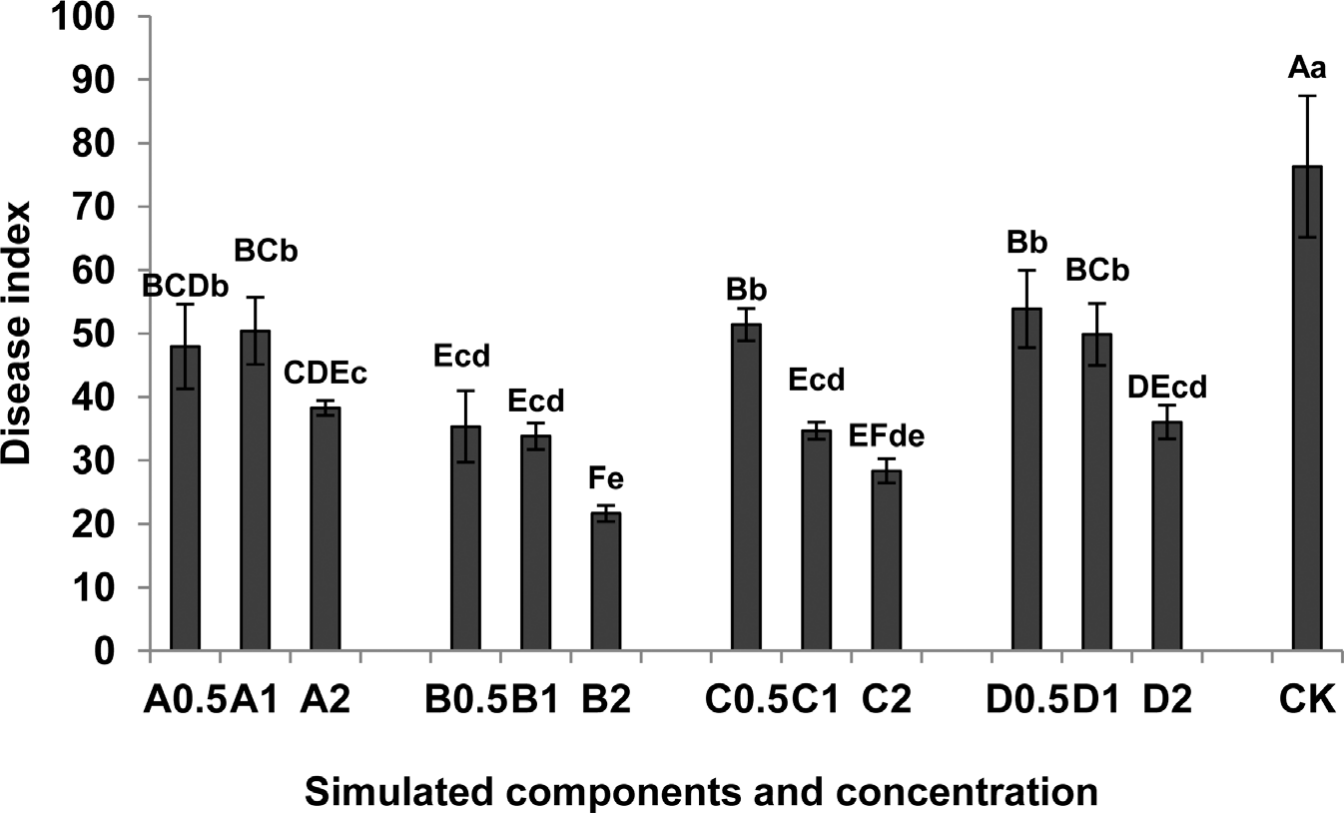
Effects of simulated components on disease resistance to *M*. *incognita* in cv. L-402. A, 2,6-Di-tert-butyl-p-cresol; B, L-ascorbyl-2,6-dipalmitate; C, dibutyl phthalate; D, dimethyl phthalate; CK: 1.0% ethanol; 0.5,0.5 mmol·L^-1^; 1,1 mmol·L^-1^; 2,2 mmol·L^-1^. Each bar represents the mean, and error bars indicate standard error of the mean from three replicates. Capital and lower case letters indicate significant group differences at the levels of 0.01 and 0.05, respectively.

The four compounds were all associated with a reduction of the disease index of L-402 tomato seedlings (Fig 6; S3 Table) which decreased as concentration increased. Seedlings irrigated with L-ascorbyl 2,6-dipalmitate (B) at the highest concentration (2 mmol·L^-1^) showed the best resistance to *M*. *incognita* with a disease index of 21.63. This was followed by dibutyl phthalate (C) at the highest concentration (2 mmol·L^-1^) where the disease index was 28.35. The disease index of above two simulated components was significantly lower than other treatments (p < 0.01).

## Discussion

### Effect of root exudates on *M*. *incognita*

Plant roots release numerous chemical compounds including amino acids, complex polysaccharides, protein and smaller, volatile, lipophilic molecules. All of these directly and indirectly influence soil organisms, such as the nematodes [32]. Extensive studies have shown that root exudates influence egg hatch of nematodes, those from nematode resistant plants have been observed to suppress the rate of egg hatch while exudates from nematode susceptible plants may stimulate egg hatch [33–35]. The current study found that root exudates from both resistant and susceptible tomato rootstock suppressed egg hatch of *M*. *incognita*. The suppression rates of root exudates from two resistant rootstocks were significantly higher than the susceptible one. The results imply that there are the differences in root exudates composition between resistant and susceptible rootstock which result in different effects on egg hatch of *M*. *incognita*. After 8 days of incubation, root exudates were diluted 2 times in distilled water. The unhatched eggs were incubated again for 7 days to check the reversibility of hatch inhibition. The result showed that inhibiting effect hadn’t been weakened obviously by the dilution of root exudates and extension of incubation time. It is further proved that the inhibition effect of root exudates on egg hatch may be sustainable.

Root exudates contribute to recognition and guiding of nematodes to plant hosts. Chemicals in root exudates attract nematodes to the roots or result in repellence, motility inhibition or even death [16]. For example, semiochemicals such as the small lipophilic molecules emitted by root exudates of tomato and rice have been found to affect both stylet thrusting and motility of the *Meloidogyne incognita* J2, with evidence that they may trigger repellent or allelopathic effects [36].The current study found that root exudates from both resistant and susceptible tomato rootstock increased significantly J2 mortality. Root exudates from Baliya (HR) had the highest relative corrected mortality of J2. As previously reported, root exudates can induce the quiescence of nematodes which is usually a reversible response in nematodes to toxic or unfavourable environmental conditions [37]. For instance, exudate from root tip of green pea (*Pisum sativum* L.) caused a loss of mobility and induced a transient and reversible quiescence in several plant-parasitic nematode species [38–39]. Similarly, in our analyses, nematode quiescence was observed both in the root exudates from Baliya (HR) and L-402 (T) at 6 h after the initial, then recovered before 18 h. In addition, the current study found that root exudates from L-402 (T) attracted J2, while root exudates from Baliya (HR) repelled them. Thus, while differences in susceptibility are generally attributed to the presence of nematode resistant genes, our work suggests that differences in root exudate composition may also be an important reason accounting for resistance to *M*. *incognita*.

### Allelopathic effects of simulated components on *M*. *incognita*

At present, few studies report identification of root exudate composition from tomato. Jia [35] analyzed the composition changes of root exudates from tomato rootstocks of varying resistance using GC-MS detection, and found that certain components including 1,2-benzenedicarboxylic acid diisooctyl ester, 2,2-methylenebis [6-(1,1-dimethylethyl) -4-methylphenol, L-(+)-ascorbic acid 2,6-dihexadecanoate, 1,2-benzenedicarboxylic acid dibutyl ester, phthalic acid and 2-benzothiazolinone et al., might correlate with tomato resistance to *M*. *incognita*, but did not attempt to verify their effects. Using the same method, we analyzed the composition changes of root exudates from Baliya (HR), RS2 (MR) and L-402 (T) before and after *M*. *incognita* inoculation. The results showed that the content of esters and phenols increased significantly in the root exudates from resistant tomato root stocks after inoculation. The relative amount of 2,6-Di-tert-butyl -p-cresol, L-ascorbyl 2,6-dipalmitate, dibutyl phthalate and dimethyl phthalate displayed the biggest changes. Because the composition changes were only induced by J2 infestation, we deduce that these chemical compounds may be closely correlated with *M*. *incognita*. It is worth noting that L-ascorbyl 2,6-dipalmitate and dibutyl phthalate were also detected in Jia’s experiment [35]. These two compounds are more likely to have an influence on nematode behavior.

This experiment verified respectively the allelopathic effect of four compounds in four concentration gradients on egg hatch of *M*. *incognita*, survival and chemotaxis of *M*. *incognita* J2, and the disease index of L-402 (T). The results showed firstly that L-ascorbyl 2,6-dipalmitate (B) suppressed most notably from the four components which may explain the observed effects of root exudates from the tomato rootstocks on egg hatch rate where the amount of L-ascorbyl 2,6-dipalmitate (B) that increased with nematode resistance in the tomato plants (T to HR) (Table 2). In addition, it was observed that dimethyl phthalate (D) at 2 mmol·L^-1^ showed the highest corrected mortality of J2, and only dibutyl phthalate (C) repelled *M*. *incognita* J2. As well, it was observed that the relative amount of dimethyl phthalate (D) and dibutyl phthalate (C) increased notably in root exudate from Baliya (HR) after inoculation (Table 2). Therefore, we deduce that these compounds may enhance tomato resistance to *M*. *incognita*. The subsequent field assay confirmed that the four compounds notably reduced the disease index of tomato cv. L-402 (T) which presented a decreasing tendency to disease as concentration increased. L-ascorbyl 2,6-dipalmitate (B) (2 mmol·L^-1^) and dibutyl phthalate (C) (2 mmol·L^-1^) showed notable resistance to *M*. *incognita*.

The results from this work can contribute to explaining the role root exudates play in tomato resistance to *M*. *incognita*. For example, in vivo an infestation of nematodes may stimulate composition change in root exudate so that the amount of esters and phenols, including the compounds L-ascorbyl 2,6-dipalmitate, dibutyl phthalate, dimethyl phthalate and 2,6-di-tert-butyl-p-cresol, increase significantly. Our work showed that these compounds suppressed egg hatch of this nematode species, increased mortality rate of J2, and also repelled J2. In practical terms, this would likely lead to a reduction in the number of J2 in soil, a subsequent decline in J2 infection rate, and enhanced tomato resistance to the nematodes *M*. *incognita*. However, our work and that of others also showed that the tomato root exudates consist of various compounds. It is likely that they have a complex interrelationship, the result of which will make it difficult to evaluate the concrete function of a single compound. Thus, it is clear that while our work makes a valuable contribution to knowledge of the mechanisms underlying tomato resistance to nematode infection, there is more research required.

## Supporting Information

S1 Fig Effects of root exudates on *M*. *incognita* egg hatch. Root exudates were diluted 2 times on the 8th day. Percentage of egg hatch was calculated at 8d and 15d after starting the assay.
(TIFF)

S2 Fig Effects of root exudates on motility of *M*. *incognita* J2. HR, MR and T represent the root exudates from three tomato strains: Baliya (highly resistant), RS2 (moderately resistant) and L-402 (highly susceptible), respectively. CK, sterilized water. Motility of J2 was evaluated by direct counts of individuals with and without active sinusoidal form and movement at 6, 12, 18, 24 and 48h after starting the assay.
(TIFF)

S1 Table Effects of simulated components on *M*. *incognita* egg hatch. ^a^ A, 2,6-Di-tert-butyl-p-cresol; B, L-ascorbyl-2,6-dipalmitate; C, dibutyl phthalate; D, dimethyl phthalate; 0.5,0.5 mmol·L^-1^; 1,1 mmol·L^-1^; 2,2 mmol·L^-1^. ^b^ Capital and lower case letters indicate significant group differences at the levels of 0.01 and 0.05, respectively.
(DOCX)

S2 Table Effects of simulated components on corrected mortality of *M*.*incognita* J2 at 24 and 48 h. ^a^ A, 2,6-Di-tert-butyl-p-cresol; B, L-ascorbyl-2,6-dipalmitate; C, dibutyl phthalate; D, dimethyl phthalate; 0.5,0.5 mmol·L^-1^; 1,1 mmol·L^-1^; 2,2 mmol·L^-1^. ^b^ Capital and lower case letters indicate significant group differences at the levels of 0.01 and 0.05, respectively.
(DOCX)

S3 Table Effects of simulated components on disease resistance to *M*. *incognita* in cv. L-402. ^a^ A, 2,6-Di-tert-butyl-p-cresol; B, L-ascorbyl-2,6-dipalmitate; C, dibutyl phthalate; D, dimethyl phthalate; 0.5,0.5 mmol·L^-1^; 1,1 mmol·L^-1^; 2,2 mmol·L^-1^; CK: 1.0% ethanol. ^b^ Capital and lower case letters indicate significant group differences at the levels of 0.01 and 0.05, respectively.
(DOCX)
