# Supplementary figures and images for: Effects of Tomato Root Exudates on Meloidogyne incognita
Source: PLoS One. 2016 Apr 29;11(4):e0154675. doi: 10.1371/journal.pone.0154675 (PMC4851295; doi:10.1371/journal.pone.0154675)

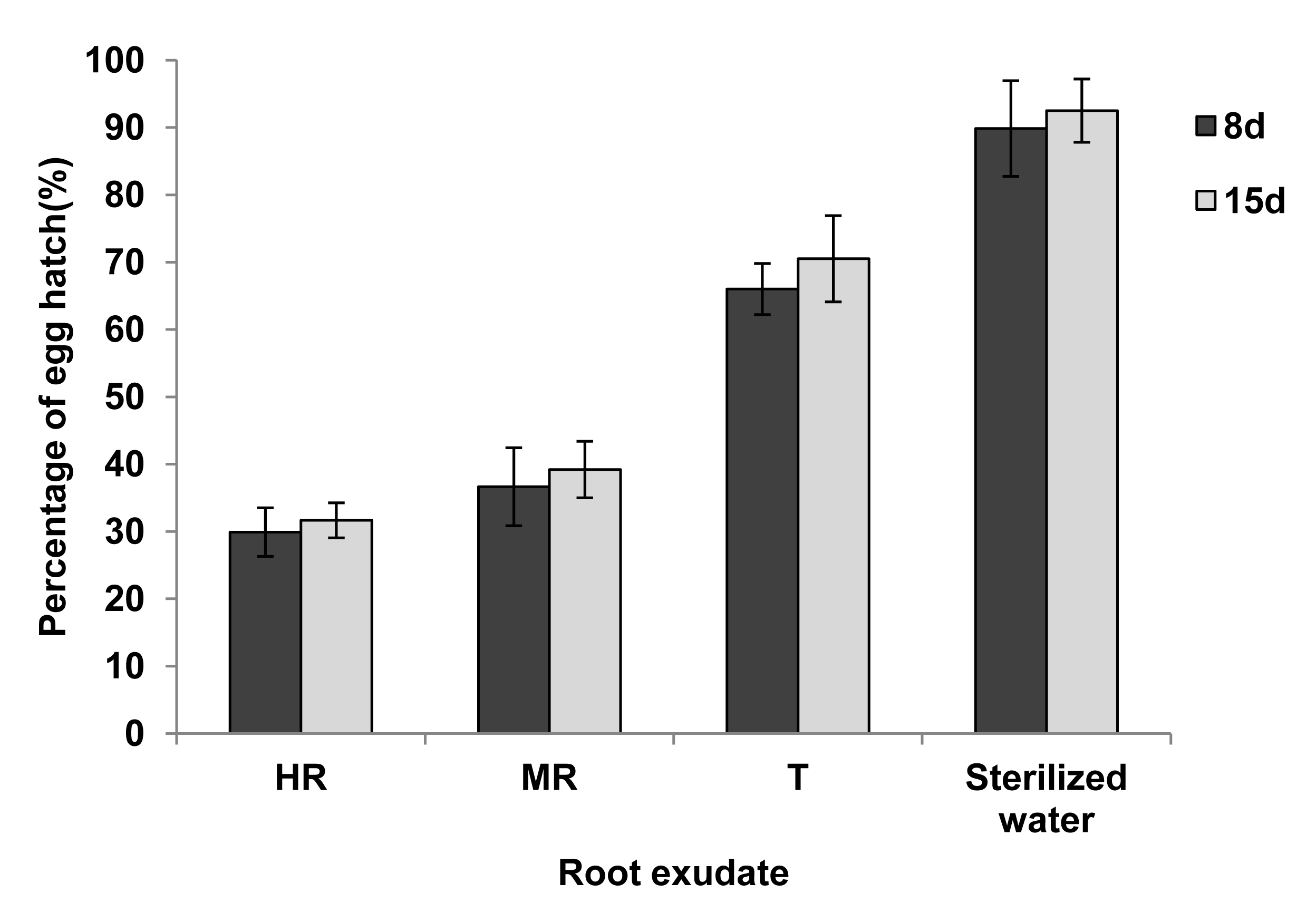

Supplement: S1 Fig — Root exudates were diluted 2 times on the 8th day. Percentage of egg hatch was calculated at 8d and 15d after starting the assay. (TIFF) [file pone.0154675.s001.tiff]

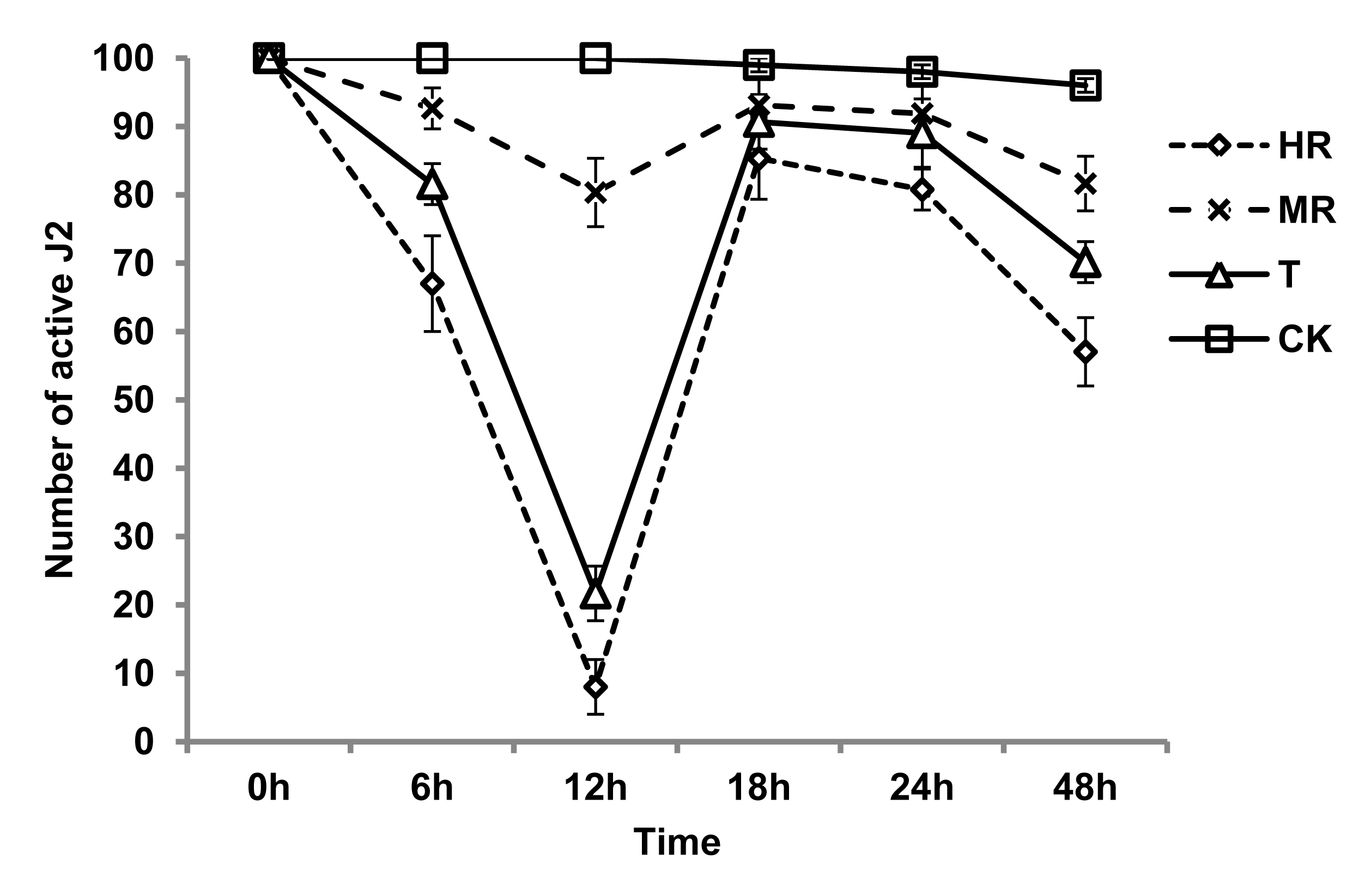

Supplement: S2 Fig — HR, MR and T represent the root exudates from three tomato strains: Baliya (highly resistant), RS2 (moderately resistant) and L-402 (highly susceptible), respectively. CK, sterilized water. Motility of J2 was evaluated by direct counts of individuals with and without active sinusoidal form and movement at 6, 12, 18, 24 and 48h after starting the assay. (TIFF) [file pone.0154675.s002.tiff]
