# Supplementary material for: Effects of Tomato Root Exudates on Meloidogyne incognita
Source: PLoS One. 2016 Apr 29;11(4):e0154675. doi: 10.1371/journal.pone.0154675 (PMC4851295; doi:10.1371/journal.pone.0154675)
Supplement: S1 Table — a A, 2,6-Di-tert-butyl-p-cresol; B, L-ascorbyl-2,6-dipalmitate; C, dibutyl phthalate; D, dimethyl phthalate; 0.5,0.5 mmol·L-1; 1,1 mmol·L-1; 2,2 mmol·L-1. b Capital and lower case letters indicate significant group differences at the levels of 0.01 and 0.05, respectively. (DOCX) [file pone.0154675.s003.docx]

**S1 Table. Effects of simulated components on *M. incognita* egg hatch.**

| **Treatment^a^** | **Relative suppression rate of**  ***M. incognita* egg hatch** | | | | **Significant differences^b^** | |
| --- | --- | --- | --- | --- | --- | --- |
|  | **Ⅰ** | **Ⅱ** | **Ⅲ** | **Average(%)** | **P < 0.01** | **P < 0.05** |
| **A0.5** | 23.89 | 30.09 | 38.05 | 30.68 | C | d |
| **A1** | 59.29 | 66.46 | 60.54 | 62.10 | B | bc |
| **A2** | 46.02 | 59.49 | 52.38 | 52.63 | B | c |
| **B0.5** | 65.82 | 72.79 | 60.38 | 66.33 | B | b |
| **B1** | 86.08 | 80.95 | 80.50 | 82.51 | A | a |
| **B2** | 76.58 | 93.20 | 88.68 | 86.15 | A | a |
| **C0.5** | 58.73 | 69.84 | 57.14 | 61.90 | B | bc |
| **C1** | 57.14 | 49.21 | 42.86 | 49.74 | B | c |
| **C2** | 73.02 | 53.97 | 52.38 | 59.79 | B | bc |
| **D0.5** | 63.49 | 58.73 | 52.38 | 58.20 | B | bc |
| **D1** | 55.56 | 52.38 | 46.03 | 51.32 | B | c |
| **D2** | 65.08 | 50.79 | 57.14 | 57.67 | B | bc |

^a^A, 2,6-Di-tert-butyl-p-cresol; B, L-ascorbyl-2,6-dipalmitate; C, dibutyl phthalate; D, dimethyl phthalate; 0.5,0.5 mmol·L^-1^; 1,1 mmol·L^-1^; 2,2 mmol·L^-1^.

^b^Capital and lower case letters indicate significant group differences at the levels of 0.01 and 0.05, respectively.
