# Supplementary material for: Effects of Tomato Root Exudates on Meloidogyne incognita
Source: PLoS One. 2016 Apr 29;11(4):e0154675. doi: 10.1371/journal.pone.0154675 (PMC4851295; doi:10.1371/journal.pone.0154675)
Supplement: S2 Table — a A, 2,6-Di-tert-butyl-p-cresol; B, L-ascorbyl-2,6-dipalmitate; C, dibutyl phthalate; D, dimethyl phthalate; 0.5,0.5 mmol·L-1; 1,1 mmol·L-1; 2,2 mmol·L-1. b Capital and lower case letters indicate significant group differences at the levels of 0.01 and 0.05, respectively. (DOCX) [file pone.0154675.s004.docx]

**S2 Table. Effects of simulated components on corrected mortality of *M.incognita* J2 at 24h and 48h.**

| **Treatment^a^** | **Corrected mortality rate of *M.incognita* J2 at 24h** | **Significant differences^b^** | | **Corrected mortality rate of *M.incognita* J2 at 48h** | **Significant differences^b^** | |
| --- | --- | --- | --- | --- | --- | --- |
|  |  | **P < 0.01** | **P < 0.05** |  | **P < 0.01** | **P < 0.05** |
| **A0.5** | 17.57 | B | bc | 27.70 | GH | g |
| **A1** | 18.92 | B | b | 57.43 | BC | bc |
| **A2** | 11.49 | BC | cde | 37.84 | EFG | ef |
| **B0.5** | 6.06 | C | e | 28.02 | GH | g |
| **B1** | 12.12 | BC | cde | 62.07 | B | b |
| **B2** | 5.30 | C | e | 48.92 | CDE | cd |
| **C0.5** | 5.41 | C | e | 25.68 | GH | g |
| **C1** | 13.51 | BC | cde | 53.38 | BCD | c |
| **C2** | 7.43 | C | de | 41.89 | DEF | de |
| **D0.5** | 5.41 | C | e | 25.00 | H | g |
| **D1** | 5.41 | C | e | 31.08 | FGH | fg |
| **D2** | 29.42 | A | a | 87.16 | A | a |

^a^A, 2,6-Di-tert-butyl-p-cresol; B, L-ascorbyl-2,6-dipalmitate; C, dibutyl phthalate; D, dimethyl phthalate; 0.5,0.5 mmol·L^-1^; 1,1 mmol·L^-1^; 2,2 mmol·L^-1^.

^b^Capital and lower case letters indicate significant group differences at the levels of 0.01 and 0.05, respectively.
