# Supplementary material for: Effects of Tomato Root Exudates on Meloidogyne incognita
Source: PLoS One. 2016 Apr 29;11(4):e0154675. doi: 10.1371/journal.pone.0154675 (PMC4851295; doi:10.1371/journal.pone.0154675)
Supplement: S3 Table — a A, 2,6-Di-tert-butyl-p-cresol; B, L-ascorbyl-2,6-dipalmitate; C, dibutyl phthalate; D, dimethyl phthalate; 0.5,0.5 mmol·L-1; 1,1 mmol·L-1; 2,2 mmol·L-1; CK: 1.0% ethanol. b Capital and lower case letters indicate significant group differences at the levels of 0.01 and 0.05, respectively. (DOCX) [file pone.0154675.s005.docx]

**S3 Table. Effects of simulated components on disease resistance to *M. incognita* in cv. L-402.**

| **Treatment^a^** | **Disease index** | | | | **Significant differences^b^** | |
| --- | --- | --- | --- | --- | --- | --- |
|  | **Ⅰ** | **Ⅱ** | **Ⅲ** | **Average** | **P < 0.01** | **P < 0.05** |
| **A0.5** | 47.21 | 41.69 | 54.98 | 47.96 | BCD | b |
| **A1** | 48.57 | 56.36 | 46.28 | 50.40 | BC | b |
| **A2** | 37.14 | 38.21 | 39.45 | 38.27 | CDE | c |
| **B0.5** | 34.29 | 30.29 | 41.38 | 35.32 | DE | c |
| **B1** | 34.29 | 35.63 | 31.56 | 33.83 | E | cd |
| **B2** | 22.86 | 20.36 | 21.66 | 21.63 | F | e |
| **C0.5** | 54.29 | 50.37 | 49.48 | 51.38 | B | b |
| **C1** | 34.29 | 36.21 | 33.57 | 34.69 | E | cd |
| **C2** | 28.57 | 26.33 | 30.14 | 28.35 | EF | de |
| **D0.5** | 52.86 | 60.39 | 48.31 | 53.85 | B | b |
| **D1** | 47.14 | 46.95 | 55.49 | 49.86 | BC | b |
| **D2** | 38.57 | 36.27 | 33.29 | 36.04 | DE | cd |
| **CK** | 74.29 | 88.32 | 66.28 | 76.30 | A | a |

^a^A, 2,6-Di-tert-butyl-p-cresol; B, L-ascorbyl-2,6-dipalmitate; C, dibutyl phthalate; D, dimethyl phthalate; 0.5,0.5 mmol·L^-1^; 1,1 mmol·L^-1^; 2,2 mmol·L^-1^; CK: 1.0% ethanol.

^b^Capital and lower case letters indicate significant group differences at the levels of 0.01 and 0.05, respectively.
